# A Comparison of a Small-Stitch Closure With a Standard Closure in Midline Laparotomy Incisions: A Prospective Comparative Study

**DOI:** 10.7759/cureus.50035

**Published:** 2023-12-06

**Authors:** Gunjot Singh Ghai, Harish S

**Affiliations:** 1 Department of General Surgery, Jagadguru Sri Shivarathreeshwara (JSS) Medical College and Hospital, Mysuru, IND

**Keywords:** incisional hernia, surgical site infection, large stitch suture, small stitch suture, midline laparotomy incision

## Abstract

Introduction

Midline incision provides a rapid and thorough approach to the abdominal cavity and, therefore, is widely employed in both emergency and elective procedures. However, midline laparotomy is associated with many post-operative complications like wound dehiscence, incisional hernia, fistula formation, and surgical site infection (SSI). The purpose of the study is to compare the complications that occurred post-operatively in association with the long and small bite suture techniques for midline laparotomy incisions.

Methodology

A prospective comparative study was carried out among 90 cases of midline laparotomies for 18 months (January 2021 to June 2022). The participants were categorized into two groups: (1) Group A, which included 45 patients, underwent small-suture abdominal fascia closure, and (2) Group B, which consisted of the remaining 45 patients, underwent large-suture abdominal midline wound closure. Patients were followed up in the hospital till the day of discharge and in the outpatient department (visit 1, within one month of surgery) for pain, wound dehiscence, and surgical site infection. Patients were called for visit 2 (six months post-surgery) and incisional hernia rates were assessed.

Results

On the numerical pain rating scale, the mean score was higher in Group B, that is, 4.1 than in Group A, which was 3.5. Wound dehiscence and incisional hernia rates were higher among Group B cases. The majority of SSIs were noted in Group B with statistically significant results.

Conclusion

Despite the lack of randomization, the results demonstrated in this study support the use of small bite sutures in comparison to long bite sutures for laparotomy closure.

## Introduction

A midline incision is frequently used in many emergency and elective surgeries due to its rapid and thorough approach to the abdominal cavity [1]. Owing to the anatomy of the abdominal wall, damage to the abdominal muscles, vessels, and nerves during a midline incision is minimal [1]. However, midline laparotomy is associated with many postoperative complications, such as fistula formation, surgical site infection (SSI), incisional hernia, and wound dehiscence [2]. SSI is reported in 15% of patients who have undergone major surgery, like midline laparotomy, which can further lead to wound dehiscence and incisional hernia [3]. These complications associated with midline laparotomies lead to morbidity, re-operation, prolonged hospital stay, and decreased quality of life [4]. Additionally, factors such as the choice of suture technique and suture material also play an important role, in association with patient factors like obesity, male sex, local wound infection, and decreased albumin levels [5]. A continuous, slowly absorbable suture material is ideal in midline laparotomies [6,7].

Furthermore, it is essential to maintain a suture length-to-wound length (SL: WL) ratio of at least 4. In the absence of this, patients are three times more likely to experience an incisional hernia [8-11]. An SL: WL ratio greater than 4 is achievable in two ways: either by placing many small stitches and reducing inter-suture distance or by taking a larger amount of tissue into stitches with greater inter-suture distance [7,10]. It is important to remember the fundamentals of wound closure, which involve quick healing with minimal strain on sutures, low infection rates, and providing strength [5,12].

According to the long stitch suture technique, the distance between the suture bite site and the wound incision should be 1 cm, and the inter-suture distance should also be 1 cm. This traditional approach of long stitch suturing includes the aponeurosis, sub-cuticular fat, and muscle within the suture [13]. If force is applied, the soft tissue gets compressed, leading to a greater amount of necrotic soft tissue and delayed wound healing [3,6,8,14]. Not only this, but the compression of soft tissue also causes the suture to cut through the tissue, leading to a separation of the aponeurotic borders, impairing collagen deposition, and resulting in a higher rate of postoperative complications [8].

In the short stitch suture technique, only the aponeurotic layer is brought into apposition, and the tension on one stitch is reduced by the addition of extra sutures. Thus, there is less slackening of sutures and reduced necrotic tissue [15]. Hence, the chances of SSI, wound dehiscence, and incisional hernia are lower [8,16]. Therefore, this study intends to compare the postoperative complications of midline laparotomy incision using the traditional long bite suture technique with the small bite suture technique.

## Materials and methods

After approval from the Institutional Ethics Committee (IEC) of Jagadguru Sri Shivarathreeshwara (JSS) Medical College and Hospital, Mysuru, Karnataka, India, with IEC reference number JSS/MC/PG/5156/2020-21, a prospective comparative study was conducted from January 2021 to June 2022. The intervention was carried out from January 1, 2021, to December 31, 2021, which was followed by a six-month follow-up period until June 30, 2022. The inclusion criteria included patients aged 18 years or older who underwent midline laparotomies, with American Society of Anaesthesiologists (ASA) status I to III, an SL:WL ratio of more than 4, patients of both genders, and patients willing to participate in the study. Patients were excluded from the study if they had a BMI greater than or equal to 30 kg/m^2^, a history of midline incision surgery, a prior incisional hernia following a midline incision, any coagulation disorders, were pregnant, or were unwilling to participate in the study.

After obtaining written informed consent, 90 patients in total were enrolled in the study based on the inclusion and exclusion criteria. The patients were divided into two groups. Each group received explanations of the surgical procedure and potential complications. In Group A, consisting of 45 patients, the abdominal fascia was closed with a small suture, where the distance between two sutures and the distance between the suture bite and midline incision was 5 mm, respectively. In Group B, which included 45 patients, a large suture was made, where the distance between two sutures and the distance between the suture bite and midline incision was 1 cm, respectively. The suture material used was USP 2/0 polydioxanone suture (PDS) Plus II in both groups.

**Figure 1:**
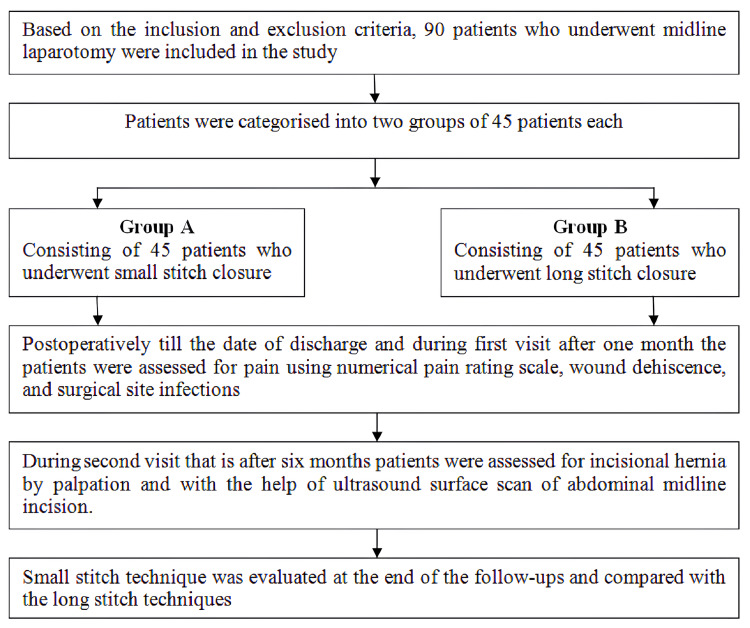
Methodological flowchart

Intraoperatively, the length of the midline incision was noted before initiating the midline laparotomy, and during the closure of the fascia, one of the aforementioned techniques was followed, and the length of the suture material was measured. The SL:WL ratio was calculated and if greater than 4, the patient was included in the study. Postoperatively, patients were clinically observed for any complications. Assessments for pain, wound dehiscence, and SSI were conducted until the day of discharge and during the first visit one month after discharge. During the second visit, six months post-discharge, incisional hernias were evaluated using ultrasound. After six months of follow-up, the proportion of postoperative complications in each group was studied. The outcomes demonstrated that postoperative complications were less in the small-stitch suturing technique compared to the large-stitch suturing technique. Statistical calculations were analyzed using IBM SPSS Statistics for Windows, Version 24 (Released 2016; IBM Corp., Armonk, New York) after data tabulation in an Excel spreadsheet (Microsoft Corporation, Redmond, Washington). T-tests were utilized for quantitative data, while chi-square tests were employed for qualitative data using SPSS and OpenEpi software. A p-value <0.05 was considered statistically significant. The procedure is illustrated in Figure 1.

## Results

A total of 90 patients were included in the study and were categorized into two groups: Group A (small-stitch suture) and Group B (long-stitch suture), each consisting of 45 patients. The age distribution of the patients showed that the majority were between 30 and 60 years old, with 26 patients in the small stitch closure group and 33 in the long stitch closure group. This was followed by those older than 60 years, comprising 15 patients in the small stitch closure group and 9 in the long stitch closure group. The group of patients younger than 30 years included 4 in the small stitch closure group and 3 in the long stitch closure group. The mean age in the small stitch closure group was 51.4±14.4 years, while in the long stitch closure group, it was 50.9±12.2 years. Gender-wise distribution among both groups showed a female preponderance, with Group A including 26 females and 19 males, and Group B consisting of 28 females and 17 males. Additionally, the mean BMI of Group A was 24.3±2.1 kg/m^2^, and for Group B, it was 24.6±1.7 kg/m^2^.

**Figure 2:**
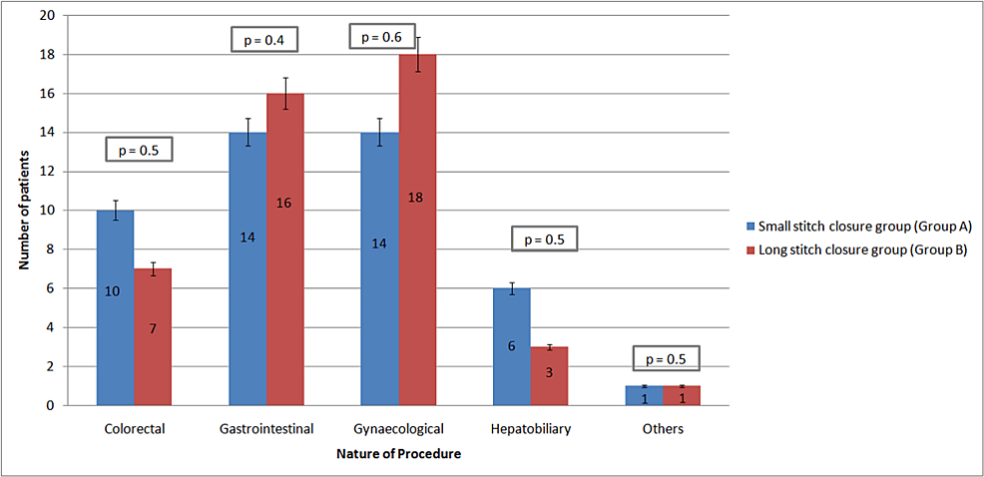
The nature of procedures in the small- and long-stitch suture groups

The nature of the most common procedures is depicted in Figure 2. Among both groups, gynecological procedures were the most common, followed by gastrointestinal, colorectal, hepatobiliary, and others.

When a comparison of various parameters between both groups was observed, the mean incision length was slightly more in Group A than in Group B, measuring 18.7 centimeters in Group A and 18.3 centimeters in Group B. However, these results were statistically insignificant (p-value = 0.3). In Group A, the mean suture length was greater, at 113 cm, compared to 90.5 cm in Group B, with statistically significant results (p-value < 0.001). Similarly, the suture length to wound length ratio was higher in Group A at 6.07, compared to 5 in Group B, again yielding statistically significant results (p-value < 0.001). Additionally, the time required for closure of the stitch was 22.2 minutes in Group A and 15.7 minutes in Group B, with statistically significant results (p-value < 0.001). Furthermore, the length of hospital stay in Group A was 8.2 days, compared to nine days in Group B, but these results were statistically insignificant (p-value = 0.3). On the numerical pain rating scale, the mean score was higher in Group B at 4.1±0.8, in comparison to Group A, which showed a mean score of 3.5±1, with statistically significant results (p-value = 0.002).

**Figure 3:**
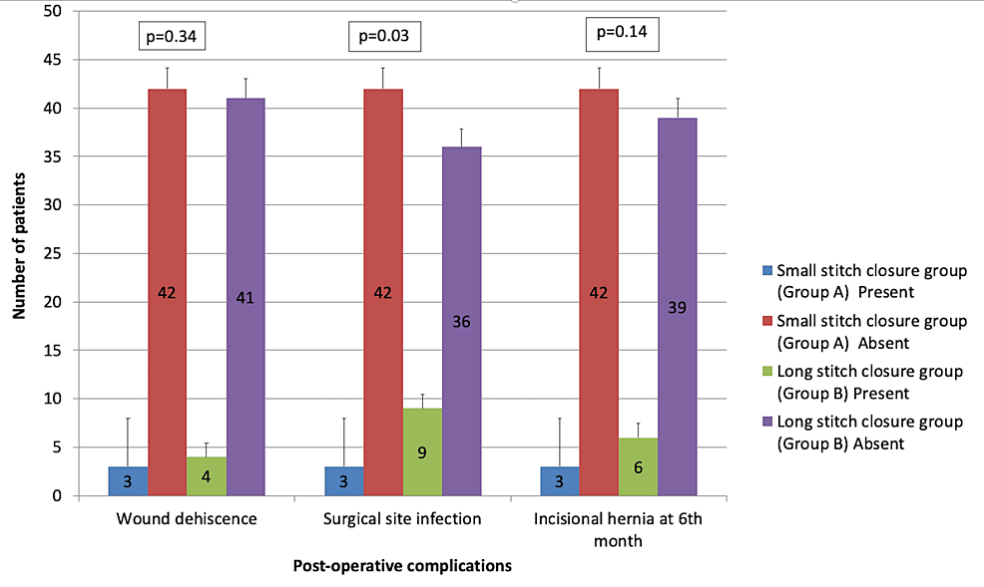
Comparison of postoperative complications between small- and long-stitch suture groups

The comparison of postoperative complications between both groups is depicted in Figure 3. More complications were seen in Group B compared to Group A.

## Discussion

The current study included a total of 90 patients who underwent midline laparotomy and were categorized into two groups of 45 patients each. Group A consisted of 45 patients in which the abdominal fascia was closed with a small stitch closure, where the distance between two sutures and the distance between the suture bite and midline incision was 5 mm, respectively. Group B included 45 patients in which a large stitch closure was performed, with the distance between two sutures and the distance between the suture bite and midline incision being 1 cm, respectively. The sample size is noticeably smaller, and the techniques used also differed in comparison to studies by Millbourn et al., who observed 356 patients with the small-bite technique and 381 with the large-bite technique; Deerenberg et al. demonstrated 276 patients with the small-bite technique and 284 with the large-bite technique; Kumar et al. with 30 in each group; deVries et al. study involving 156 patients with the small-bite technique and 191 with the large-bite technique; and Sharma et al. with 50 patients in each group [5,8,14,17,18].

For the small bite technique, the mean age of patients in Group A was 51.4±14.4 years, and for the large bite technique, it was 50.9±12.2 years in Group B. In comparison, Millbourn et al. demonstrated the mean age for the small bite technique of 65 years and 64 years for the large bite technique. Similarly, Deerenberg et al. reported a mean age of 62 years and 63 years in small and large bite techniques, respectively [8,14]. Additionally, deVries et al. reported the mean age for small and large bite techniques as 69 years and 68 years, respectively [18]. Furthermore, Sharma et al. reported the mean age of the individuals in the small and large bite techniques as 46.34 years and 45.78 years, respectively [5]. In the present study, when gender distribution among both groups was compared, a female preponderance was observed, which was similar to previous studies [5,14,17,18]. In contrast, the study done by Millbourn et al. demonstrated male preponderance in both suture techniques [8].

The present study illustrated a comparison of the small bite technique and the large bite technique in various procedures, but the most commonly involved procedure was gynecological, followed by gastrointestinal, colorectal, hepatobiliary, and others. In contrast, Millbourn et al. and Deerenberg et al. studied the small bite versus large bite technique mainly in gastrointestinal and colorectal surgeries, respectively [8,14]. The mean incisional length parameter in the present study was 18.7 cm for the small-bite technique and 18.2 cm for the large-bite technique. Similarly, in a study by Deerenberg et al., in both groups, the mean incision length was 22 cm [14]. The mean suture length in the present study was 113 cm in the small bite technique group and 90.5 cm in the large bite technique group. Likewise, in studies by Deerenberg et al. and Sharma et al., it was 110 cm and 115.7 cm in the small-bite technique, respectively, and 95 cm and 88.82 cm in the large-bite technique, respectively [5,14]. Hence, it was observed that in the case of Group A patients, the mean suture length was greater in comparison to Group B patients.

The ratio of SL:WL in the present study was 6.07 in the small-bite technique group and 5 in the large-bite technique group. Similarly, in a study by Deerenberg et al., it was 5 in the small-bite technique and 4.3 in the large-bite technique [14]. In contrast, Millbourn et al. and Kumar et al. reported ratios of 5.7 and 5.2 in the small-bite technique, respectively, and 6.4 each in the large-bite technique, respectively [8,17]. Additionally, when the mean time for closure was compared in minutes between both groups in the small-bite technique, it was 22.2 minutes, and in the large-bite technique, it was 15.7 minutes. Similarly, in studies by Millbourn et al., Deerenberg et al., Kumar et al., and Sharma et al., compared to large stitch groups, the small stitch group's closure time was longer [5,8,14,17]. Therefore, the aforementioned findings showed that the small-bite technique group's suture closure time was longer than that of the large-bite technique group.

Furthermore, postoperative complications such as wound dehiscence, SSI, and incisional hernia were reported, for which the present study found wound dehiscence in 6.6% of patients in the small-bite technique and 8.8% of patients in the large-bite technique, demonstrating that wound dehiscence was more common in the large-bite technique group compared to the small-bite technique group. Similarly, surgical site infection was more observed in the large-bite technique group (20%) compared to the small-bite technique group (6.6%), which correlates well with the results of previous studies [5,8,14,17-19]. Another complication, incisional hernia, was assessed after 6 months of follow-up and was reported more commonly in the large-bite technique group (13.3%) than in the small-bite technique group (6.6%). These results were congruent with previous studies that reported more cases of incisional hernia in the large-bite technique group than in the small-bite technique group.

The strengths associated with the study involve a comparison of short- versus long-bite techniques, and the evidence is too strong in favor of the application of the small-bite technique; therefore, every comparative study is a contribution to the evidence base. Along with postoperative complications, various parameters have been compared in this study, which involved SL:WL ratio, incision length, suture length used, hospital stay, and closure time. The study consisted of certain limitations, which included a small sample size of 90 patients, which may not be representative of the entire population, a single-center study, long-term follow-up for incisional hernia was not performed, only cases undergoing elective laparotomy were included, and there may be a chance of randomization bias.

## Conclusions

Abdominal surgeries are among the major surgeries performed with higher incidences of morbidity and mortality. Thus, due care should be taken to avoid complications. The present study showed that surgical site infections, wound dehiscence, and incisional hernias were seen more frequently in the large-bite group compared to the small-bite group. Previous recommendations favoring large stitches should be revised to advocate for small stitches. Such techniques should be incorporated into standard treatment guidelines and thus followed by surgeons. For this, they should be trained in small-bite incision techniques, leading to minimal post-surgical complications.
